# Cardioprotective Effects of Puerarin-V on Isoproterenol-Induced Myocardial Infarction Mice Is Associated with Regulation of PPAR-Υ/NF-κB Pathway

**DOI:** 10.3390/molecules23123322

**Published:** 2018-12-14

**Authors:** Xuguang Li, Tianyi Yuan, Di Chen, Yucai Chen, Shuchan Sun, Danshu Wang, Lianhua Fang, Yang Lu, Guanhua Du

**Affiliations:** 1 State Key Laboratory of Bioactive Substances and Functions of Natural Medicines, Institute of Materia Medica, Chinese Academy of Medical Sciences and Peking Union Medical College, Beijing 100050, China; 13681509820@163.com (X.L.); yuantianyi@imm.ac.cn (T.Y.); 2 Beijing Key Laboratory of Drug Target Identification and Drug Screening, Institute of Materia Medica, Chinese Academy of Medical Sciences and Peking Union Medical College, Beijing 100050, China; chendi@imm.ac.cn (D.C.); maxchenyucai@163.com (Y.C.); sunsc@imm.ac.cn (S.S.); wangdanshu@imm.ac.cn (D.W.); 3 Beijing Key Laboratory of Polymorphic Drugs, Institute of Materia Medica, Chinese Academy of Medical Sciences and Peking Union Medical College, Beijing 100050, China; luy@imm.ac.cn

**Keywords:** puerarin-V, myocardial infarction, anti-inflammation, anti-apoptosis, PPAR-Υ, NF-κB

## Abstract

Puerarin is a well-known traditional Chinese medicine which has been used for the treatment of cardiovascular diseases. Recently, a new advantageous crystal form of puerarin, puerarin-V, has been developed. However, the cardioprotective effects of puerarin-V on myocardial infarction (MI) heart failure are still unclear. In this research, we aim to evaluate the cardioprotective effects of puerarin-V on the isoproterenol (ISO)-induced MI mice and elucidate the underlying mechanisms. To induce MI in C57BL/6 mice, ISO was administered at 40 mg/kg subcutaneously every 12 h for three times in total. The mice were randomly divided into nine groups: (1) control; (2) ISO; (3) ISO + puerarin injection; (4–9) ISO + puerarin-V at different doses and timings. After treatment, cardiac function was evaluated by electrocardiogram (ECG), biochemical and histochemical analysis. In vitro inflammatory responses and apoptosis were evaluated in human coronary artery endothelial cells (HCAECs) challenged by lipopolysaccharide (LPS). LPS-induced PPAR-Υ/NF-κB and subsequently activation of cytokines were assessed by the western blot and real-time polymerase chain reaction (PCR). Administration of puerarin-V significantly inhibits the typical ST segment depression compared with that in MI mice. Further, puerarin-V treatment significantly improves ventricular wall infarction, decreases the incidence of mortality, and inhibits the levels of myocardial injury markers. Moreover, puerarin-V treatment reduces the inflammatory milieu in the heart of MI mice, thereby blocking the upregulation of proinflammatory cytokines (*TNF-α*, *IL-1β* and *IL-6*). The beneficial effects of puerarin-V might be associated with the normalization in gene expression of PPAR-Υ and PPAR-Υ/NF-κB */ΙκB-α/ΙΚΚα/β* phosphorylation. In the in vitro experiment, treatment with puerarin-V (0.3, 1 and 3 μM) significantly reduces cell death and suppresses the inflammation cytokines expression. Likewise, puerarin-V exhibits similar mechanisms. The cardioprotective effects of puerarin-V treatment on MI mice in the pre + post-ISO group seem to be more prominent compared to those in the post-ISO group. Puerarin-V exerts cardioprotective effects against ISO-induced MI in mice, which may be related to the activation of PPAR-γ and the inhibition of NF-κB signaling in vivo and in vitro. Taken together, our research provides a new therapeutic option for the treatment of MI in clinic.

## 1. Introduction

Ischemic heart disease (IHD), also known as coronary artery disease (CAD), is a class of heart diseases, such as angina pectoris, myocardial infarction (MI), ischemic heart failure, and arrhythmia. IHD is prevalent worldwide and MI is the leading cause of deaths and disabilities [1]. MI is an acute condition of myocardial necrosis that is caused by the imbalance between coronary blood supply and myocardial demand [2]. Previous studies have revealed that MI results from a complex set of pathological processes, including an increase in free radical formation, apoptosis, inflammatory cell infiltration, irreversible DNA damage and other possible mechanisms [3,4]. At present, clinical therapies for IHD are palliative in the sense that the clinical symptoms are only improved by increasing blood perfusion, reducing myocardial oxygen consumption. However, the effects of current therapies are strictly limited, and also accompanied with obvious side effects. Therefore, it is necessary to search for a novel compound with lower toxicity for the prevention and treatment of IHD.

It is widely accepted that isoproterenol (ISO) injection can readily induce MI in mice. The anti-inflammatory activity is one of the key mechanisms of anti-MI efficacy [1]. MI-mediated adverse reactions are characterized by activation of many cellular signaling molecules such as NF-κB and PPAR-Υ. NF-κB is a key regulator in the condition of inflammation and apoptosis. In MI, phosphorylation of NF-κB initiates the intracellular signaling cascade, and finally induces pro-inflammatory cytokines such as TNF-α, IL-6, IL-1β as well as other inflammation-related proteins that induce various pathophysiological changes [5]. PPAR-γ, as a member of nuclear receptors, could directly combine with p65/p50 to reduce the DNA-binding activity of NF-κB, which suggests that the anti-inflammatory properties of the PPAR-γ ligand may be related to the regulation of NF-ΚB activation [6]. Upregulation of PPAR-γ by curcumin treatment has shown cardioprotective effects both in vivo and in vitro [7,8]. PPAR-γ is considered to have a crucial role in regulating inflammatory signaling and cytokines production. It was reported that the expression and activation of PPAR-γ block inflammation induced by ISO [9]. Apoptosis is an important contributor to the formation of MI, especially after pro-inflammatory cytokines expression. PPAR-γ has an anti-apoptotic effect herein by modulation of caspase 3 [10]. Therefore, regulation of PPAR-γ and NF-ΚB signaling pathways might be a promising strategy for the treatment of MI.

**Figure 1:**
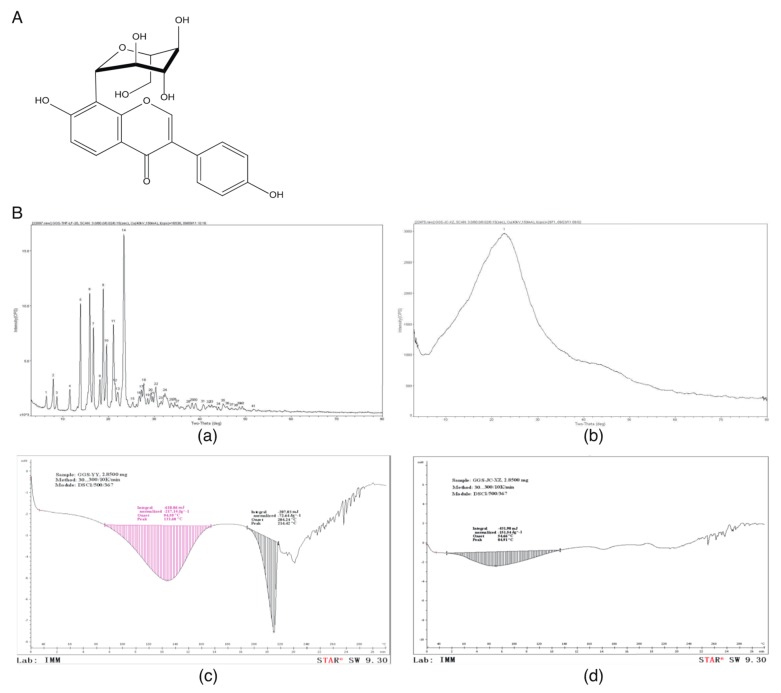
Structure of puerarin-V and differences in crystal characterization between puerarin-V and puerarin. (**A**) The chemical structure of puerarin-V. (**B**) Representative images of powder X-ray diffraction (PXRD) of puerarin-V (**a**) and puerarin (**b**); representative images of differential scanning calorimetry (DSC) of puerarin-V (**c**) and puerarin (**d**).

Puerarin (7,4′-dihydroxy-8-β-d-glucosylisoflavone, C_21_H_20_C_9_), which is extracted from Chinese herb Kudzu root, is widely used as a clinical adjuvant medicine to treat cardiovascular diseases. Puerarin injection has been widely used in the treatment of IHD clinically, such as coronary heart disease, angina pectoris and MI. Therefore, puerarin injection is also utilized as a positive control to evaluate the effects against MI in the present studies. Among flavonoids, puerarin is one of the most bioactive compounds and exhibits many pharmacological effects including antioxidant, anti-inflammation, vasodilation, and improvement of endothelial function through different mechanisms of actions [11]. Accumulating studies found that puerarin may inhibit cardiac fibrosis and accelerate cardiac angiogenesis to improve cardiac function in MI animals [12,13,14]. Due to the low solubility and poor bioavailability of puerarin, several modified crystal forms of puerarin were developed by Beijing Key Laboratory of Polymorphic Drugs of the Institute of Materia Medica of the Academy of Medical Sciences. Among the five new crystals of puerarin, puerarin-V has a better absorption and a higher plasma drug concentration than the other four crystal forms. Because of the improved features compared to original puerarin, a patent has been approved for puerarin-V. The molecular formula and chemical structure of puerarin and puerarin-V are the same. However, they are different in crystal forms with different arrangements or conformations. These polymorphic forms of puerarin change its physicochemical properties such as solubility and stability, which can significantly influence its bioavailability and toxicity. For this, the chemical structure of puerarin-V and the differences in crystal characteristics between puerarin-V and puerarin are shown in Figure 1.

In this study, we explored the cardioprotective effects of puerarin-V in an animal of MI induced by ISO. The anti-inflammatory mechanism of puerarin-V was further investigated by LPS-stimulated human coronary artery endothelial cells (HCAECs) and the protective effects of puerarin-V on HCAECs were assessed. Simultaneously, we compared the effects of puerarin-V treatment between the preventive administration and therapeutic administration in MI mice. Our extensive in vivo and in vitro studies reveal the potential therapeutic effects of puerarin-V against MI and provide a new therapeutic option for the treatment of MI in clinic.

## 2. Results

### 2.1. Puerarin-V Improved Cardiac Function and Suppressed Mortality in the MI Mice

**Figure 2:**
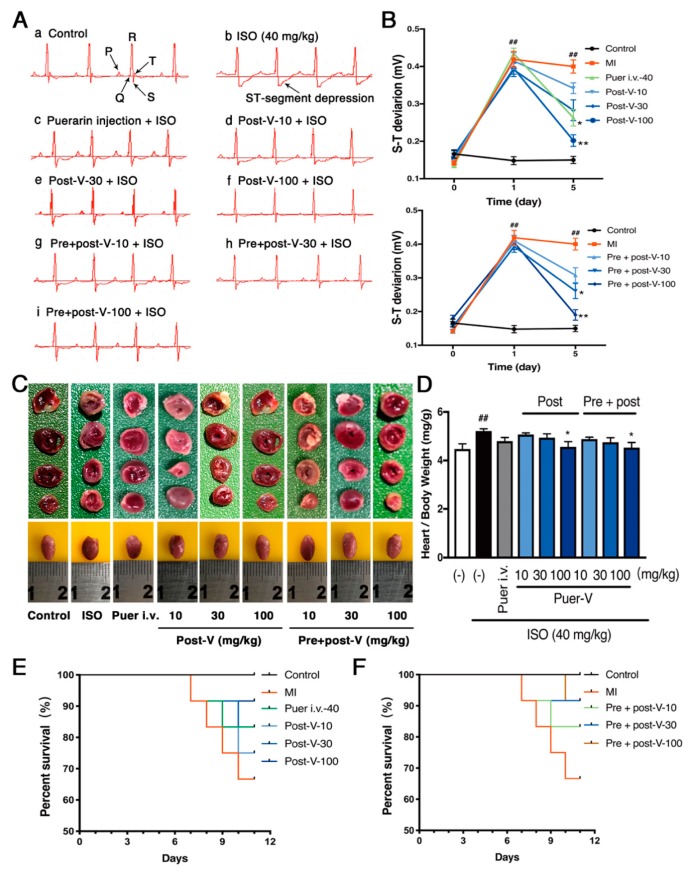
Cardioprotective effects of puerarin-V in the MI mice. (**A**) Representative images of ECG. (a)–(i) correspond to groups: (a) control group; (b) model group at day 5 after the MI induction; (c) puer i.v.-40 mg/kg group at day 5 after the MI induction; (d), (e) and (f): post-puerarin-V groups (10, 30, and 100 mg/kg, respectively) at day 5 after the MI induction; (g), (h) and (i): pre + post-puerarin-V groups (10, 30, and 100 mg/kg, respectively) at day 5 after the MI induction. (**B**) Effects of puerarin-V on ST segment deviation (mV) of MI mice. (**C**) TTC-stained heart slices arranged in order and representative images of heart samples from the indicated groups of mice are shown in the photos. The white area represents infarcted tissue. (**D**) The heart wet weight/body weight ratio in each group. (**E**,**F**) The survival rate of MI mice and normal mice in each group. All mice were observed for mortality until day 6. Data are shown as mean ± SEM (n = 8–10). ^##^ *p* < 0.01 vs. control group. * *p* < 0.05, and ** *p* < 0.01 vs. model group.

Electrocardiogram (ECG) is the process of recording the electrical activity of the heart. To test whether MI was successively induced by ISO administration in the mice, we measured ECG in each mouse treated with ISO over the time course. There was no difference of ECG between groups 24 h before MI was induced. However, the ECG showed marked ST-segment depression in the ISO-treated mice at day 1, which was gradually stabilized at day 2 (Figure 2A). In addition, ST segments and T waves also showed depression in the ISO-treated mice at day 1 (Figure 2B). To investigate the cardioprotective effects of puerarin-V, we treated mice with puerarin-V at different doses and timings, and puerarin injection was used as a positive control. The results showed that the typical ECG abnormal changes were reversed by the treatment of puerarin injection, post-treatment of puerarin-V (post-V) or pre- and post-treatment of puerarin-V (pre + post-V) at day 5 after the MI induction. Moreover, the effects on ECG patterns of post-V and pre + post-V groups at the dose of 100 mg/kg were comparable in puerarin injection treatment. There was no difference observed between post-V and pre + post-V.

Next, the extent of myocardial infarctions was assessed by (2,3,5-triphenyltetrazolium chloride) TTC staining. As shown in Figure 2C, the whole heart tissues of control group showed no infarction, while the MI mice showed an enlarged ventricular cavity and extensive infarction in the ventricular apical area. Treatment with puerarin-V (100 mg/kg) decreased the heart index (Figure 2D), improved ventricular wall infarction, and reduced ventricular cavity. We next investigated the survival rate after MI with or without puerarin-V treatment. As shown in Figure 2E,F, after three consecutive injections of ISO, approximately 33.3% of the mice in the model group (MI) died between day 1 and day 6. While the post-ISO group treated with puerarin-V (10, 30 and 100 mg/kg) decreased the mortality rate (25%, 16.7%, and 8.3%, respectively), and the mortality rates of pre- and post-ISO group were 16.7%, 8.3%, and 8.3%, respectively. These results illustrated that puerarin-V treatment improved cardiac function and inhibited MI mortality.

### 2.2. Puerarin-V Treatment Suppressed the Myocardial Inflammation and Necrosis in the MI Mice

**Figure 3:**
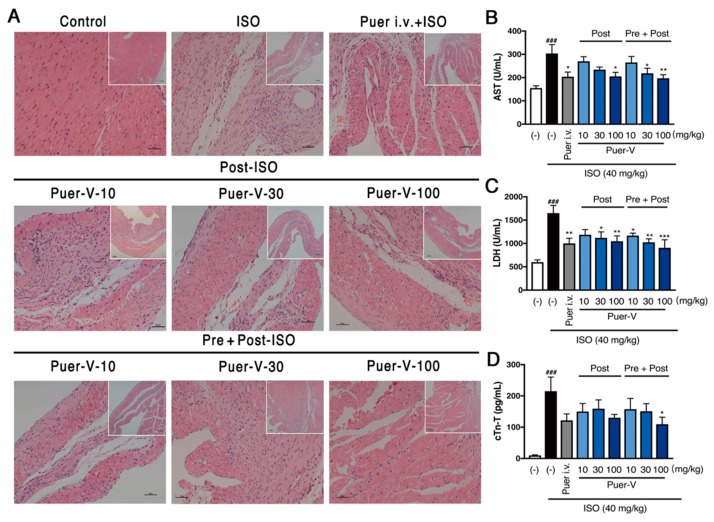
Puerarin-V treatment suppressed myocardial inflammation and reduced myocardial cell damage in the MI mice. (**A**) Representative images of H&E staining of right ventricular cardiomyocytes in different groups. Small images were 100×, and the big images were 200×. Scale bar = 100 μm. (**B**–**D**) The serum levels of AST, LDH and cTn-T. Data are shown as mean ± SEM (*n* = 6). ^###^ *p* < 0.001 vs. control group. * *p* < 0.05, ** *p* < 0.01, and *** *p* < 0.001 vs. model group.

The myocardial lesions of MI were mainly localized in the endocardial and middle layers of the myocardium. ISO-induced MI was characterized by infiltration of inflammatory cells with multiple localized necrosis. Puerarin-V treatment mildly ameliorated the pathological exacerbation of myocardial tissue as implied by (hematoxylin and eosin) H&E staining (Figure 3A). The size of patchy lesions of mice hearts in the post-ISO group and pre + post-ISO group were smaller, and the numbers of inflammatory cells were reduced as compared to those in the MI group.

The levels of myocardial injury markers released into the serum are commonly used to diagnose and monitor MI for the clinical, and therefore the leakage of enzyme is considered to be a marker of myocardial cell damage [15,16]. Therefore, aspartate aminotransferase (AST), lactate dehydrogenase (LDH) and cardiac troponin T (cTn-T) were detected in serum of control and experimental mice. As described in our published work [17], mice treated with ISO progressively increased in the levels of AST, LDH and cTn-T compared with the control group, indicating ISO successfully induced acute MI. Oral administration with puerarin-V almost restored all the ISO-induced alterations of AST, LDH and cTn-T. The positive control also suppressed the increased level of enzymes induced by ISO treatment (Figure 3B–D). Moreover, there was a decrease noticed in the levels of these marker enzymes in the plasma of pre + post-ISO group as compared to that of post-ISO group. Thus, it appears that puerarin-V could attenuate the cell injury as potent as the positive drug.

### 2.3. Puerarin-V Alleviated Inflammation Injury in the MI Mice

Since we observed a marked protection against ISO-induced inflammatory cells infiltration estimated by H&E staining, we set out to assess whether puerarin-V has an effect on inflammation in MI mice. We then performed the immunohistochemical staining of tissue sections with primary antibody CD68, and the gene transcripts and expression of proinflammatory cytokines including IL-6, TNF-α, and IL-1β in the heart.

**Figure 4:**
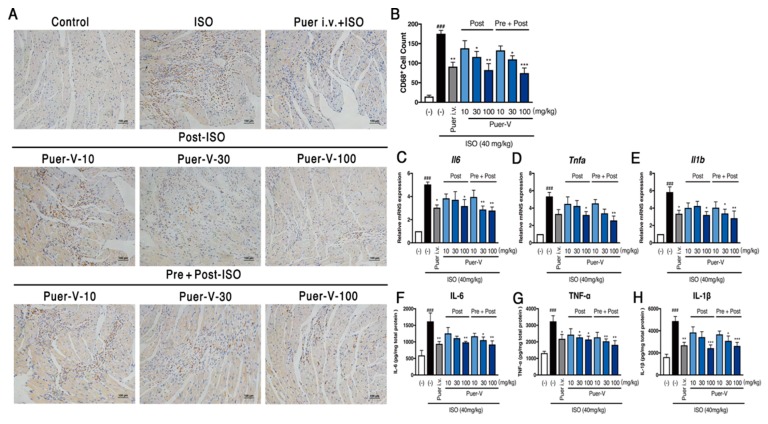
Puerarin-V reduced myocardial inflammation in the heart of MI mice. (**A**) Immunohistochemical analysis of macrophage marker CD68 in heart cross sections. The original magnification of the images was 200×. The scale bar is 100 μm. (**B**) The percent of CD68^+^ cells (*n* = 4). (**C**–**E**) The mRNA levels of IL-6, TNF-α, and IL-1β (*n* = 6). (**F**–**H**) The quantitative analyses of IL-6, TNF-α, and IL-1β by ELISA (*n* = 6). Data are shown as mean ± SEM. ^###^ *p* < 0.001 vs. control group. * *p* < 0.05, ** *p* < 0.01, and *** *p* < 0.001 vs. model group.

CD68 is considered to be a macrophage molecular marker [18]. The immunohistochemical results showed that the number of CD68^+^ cells in the model group was significantly increased (*p* < 0.001), and puerarin-V treatment reduced the number of CD68^+^ cells in a dose-dependent manner (Figure 4A,B). In addition, the proinflammatory cytokines expression was significantly upregulated in the MI group, while puerarin-V downregulated the proinflammatory cytokines on both mRNA and protein levels compared with those in the MI group (Figure 4C–H). In general, the prior administration of puerarin-V at the dose of 100 mg/kg obviously reduced the ISO-induced inflammation injury and decreased the level of the evaluated parameters. Collectively, our results suggested that puerarin-V suppressed ISO-induced myocardial inflammation to ameliorate the impaired myocardium.

### 2.4. Puerarin-V Inhibited Myocardial Apoptosis in the MI Mice

**Figure 5:**
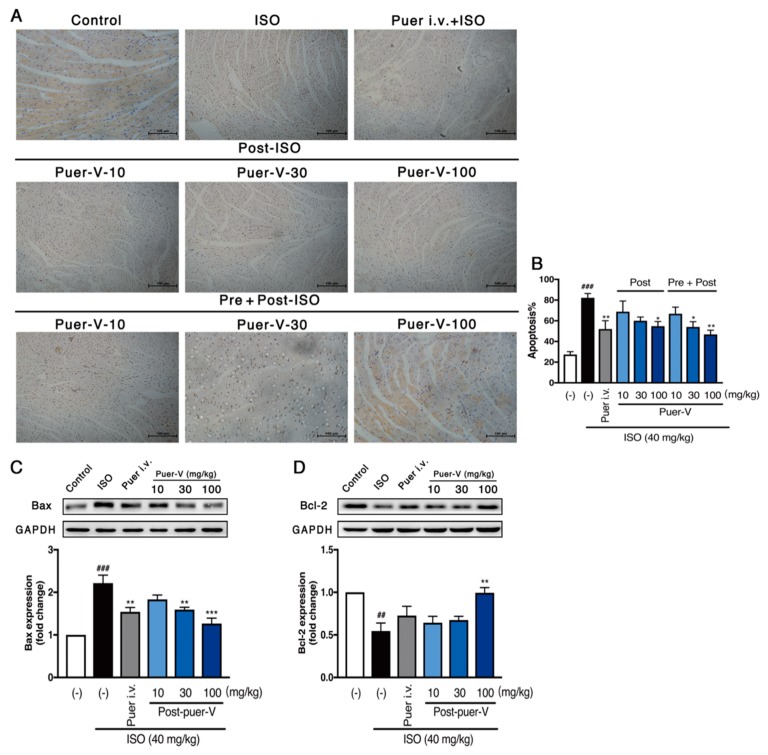
Puerarin-V inhibited ISO-induced apoptosis in the heart of MI mice. (**A**) The TUNEL-positive cells were shown by the immunohistochemistry method. The original magnification of the images was 200×. The scale bar is 100 μm. (**B**) The percent of TUNEL-positive cells (*n* = 4). (**C**,**D**) Western blot analyses for Bax and Bcl-2 expression, and normalization of these two expression by the expression of glyceraldehyde-3-phosphate dehydrogenase (GAPDH) (*n* = 6). Data are shown as mean ± SEM. ^##^ *p* < 0.01, and ^###^ *p* < 0.001 vs. control group. ** *p* < 0.01, and ***p < 0.001 vs. model group.

ISO-induced apoptosis of cardiomyocytes is a major contributor to the formation of MI, especially after proinflammatory cytokines expression. To test the effects of puerarin-V on the regulation of apoptosis induced by ISO, terminal deoxynucleotidyl transferase dUTP nick end labeling (TUNEL) and western blot were performed. As depicted in Figure 5A,B, ISO significantly increased the rate of TUNEL-positive cells represented as a percentage of the total nuclei (82.2 ± 4.14%), while the post-ISO group treated with puerarin-V (10, 30, and 100 mg/kg) decreased the number of TUNEL-positive cells (68.8 ± 10.36%, 62 ± 4.7%, and 54.8 ± 4.52%, respectively), and consistent with the trend of the pre + post-ISO group (66.8 ± 6.43%, 54 ± 5.14%, and 46.8 ± 4.08%, respectively). The results illustrated that puerarin-V inhibited the ISO-induced cell apoptosis in a dose-dependent fashion.

Additionally, the expression of Bcl-2 associated x (Bax) protein was significantly upregulated in the model group, compared to that in the control group (Figure 5C), an effect that was attenuated by treatment with 30 or 100 mg/kg puerarin-V (*p* < 0.01, and *p* < 0.001, respectively). Moreover, B-cell lymphoma-2 (Bcl-2) protein expression in heart tissue as an indicator of anti-apoptosis exhibited an opposite effect compared with that of apoptotic biomarker among the groups. As illustrated in Figure 5D, the Bcl-2 expression decreased in the model group compared with that in the control group, and the tendency could be reversed by puerarin-V treatment (100 mg/kg, *p* < 0.01). These results uncovered that puerarin-V prominently inhibited apoptosis induced by ISO in the MI mice.

### 2.5. Puerarin-V Attenuated ISO-Induced Inflammation in the MI Mice Associated with Upregulation of PPAR-γ Expression and Inhibition of NF-κB Phosphorylation

**Figure 6:**
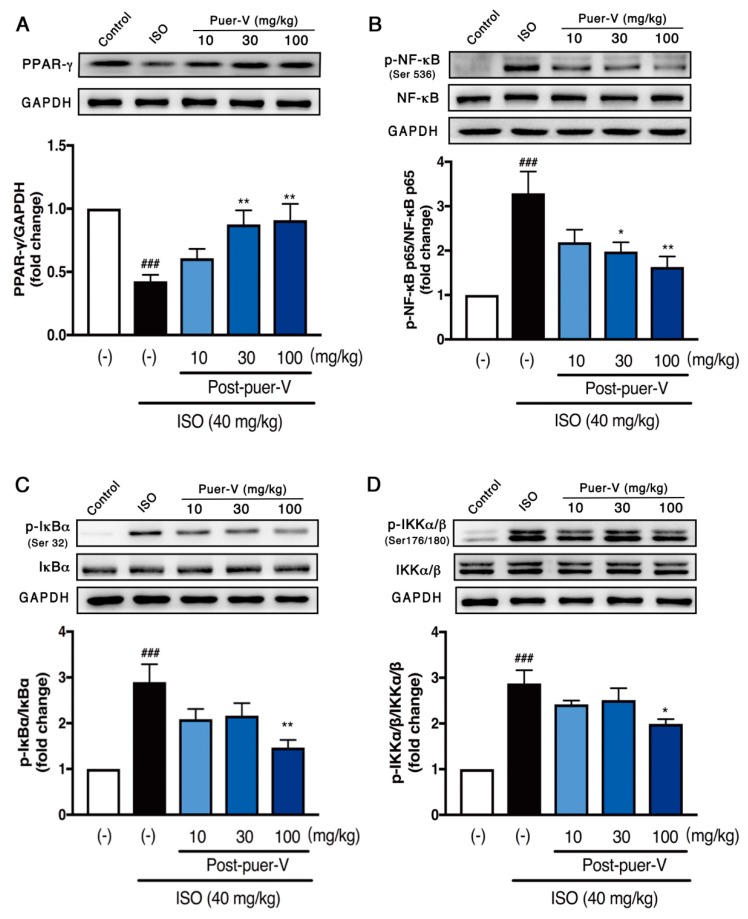
Puerarin-V attenuated ISO-induced inflammation in the MI mice associated with the PPAR-γ/NF-κB pathway: (**A**) PPAR-γ; (**B**) p-NF-κB/NF-κB; (**C**) p-IκB-α/IκB-α; and (**D**) p-IKKα/β/IKKα/β. Relative density analysis of the protein bands was shown by the western blot with GAPDH as control. Data are shown as mean ± SEM (*n* = 6). ^###^ *p* < 0.001 vs. control group. * *p* < 0.05, and ** *p* < 0.01 vs. model group.

The role of PPAR-γ has been demonstrated to be involved in the modulation of NF-κB transcriptional activity and inflammation [19]. We investigated whether the anti-inflammatory activity of puerarin-V in MI mice involves NF-κB activation and is modulated by PPAR-γ. PPAR-γ, NF-κB, IκB-α and IKKα/β protein expression were determined by the western blot. It suggested that PPAR-γ expression was downregulated after MI injury. Treatment with puerarin-V at a dose of 30 or 100 mg/kg significantly augmented the PPAR-γ expression (Figure 6A). NF-κB is the downstream pathway of PPAR-γ and involved in the MI-induced inflammation. In comparison to the control group, the model group showed a significant (*p* < 0.001) increase in NF-κB, IκB-α and IKKα/β phosphorylation. Treatment with puerarin-V significantly suppressed the activated p-NF-κB, p-IκB-α, and p-IKKα/β expression (Figure 6B–D). Collectively, puerarin-V exerts cardioprotective effects by suppressing ISO-induced inflammation associated with upregulation of PPAR-γ expression and inhibition of NF-κB phosphorylation in the MI mice.

### 2.6. Puerarin-V Protected Coronary Artery Endothelial Cells Function against LPS-Induced Inflammation Associated with Upregulation of PPAR-γ Expression and Inhibition of NF-κB Phosphorylation

**Figure 7:**
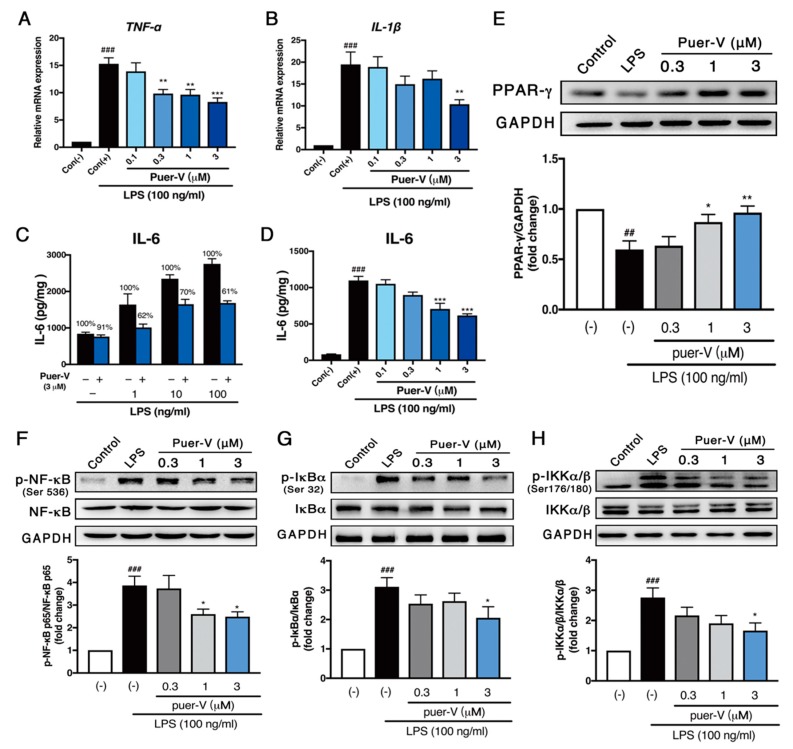
Puerarin-V attenuated LPS-induced inflammation in HCAECs associated with the PPAR-γ/NF-κB pathway. (**A**,**B**) The mRNA levels of TNF-α and IL-1β. (**C**) Confluent cultures of HCAEC were preincubated for 2 h with puerarin-V (3 μM). Thereafter, different concentrations of LPS were added and cells were cultivated for 24 h. The black bars show the cultures without puerarin-V treatment; the blue bars show the puerarin-V treated cultures. (**D**) Confluent cultures of HCAEC were preincubated for 2 h with different amounts of puerarin-V (μM). Thereafter, LPS (100 ng/ml) was added and cells were cultivated for 24 h. (**E**) PPAR-γ expression. (**F**) p-NF-κB/NF-κB expression. (**G**) p-IκB-α/IκB-α expression. (**H**) p-IKKα/β/IKKα/β expression. Relative density analysis of the protein bands was shown by the western blot with GAPDH as control. Data are shown as mean ± SEM (*n* = 4–6). ^##^ *p* < 0.01, and ^###^ *p* < 0.001 vs. control group. * *p* < 0.05, ** *p* < 0.01, and *** *p* < 0.001 vs. model group.

We next focused on the changes in endothelial inflammatory cytokines to elucidate the protective effects of puerarin-V on endothelial cells. As expected, stimulation with LPS (100 ng/mL) for 24 h progressively increased the mRNA levels of TNF-α and IL-1β (Figure 7A,B) in HCAECs. Pre-incubation with different concentrations (0.3–3 μM) puerarin-V for 2 h could attenuate this increase in a dose-dependent manner. In addition, pre-incubation with puerarin-V (3 μM) with different concentrations of LPS reduced both basal and LPS-induced expression of IL-6 (Figure 7C). Then, pre-incubation of HCAECs with different concentrations of puerarin-V (0.1–3 μM) reduced the production of IL-6 in a concentration-dependent manner (Figure 7D), indicating that puerarin-V can inhibit LPS-induced HCAECs inflammatory activity.

In accordance with the results in vivo, we found that PPAR-γ protein level was significantly decreased in LPS-treated HCAECs, and the protein levels of *p*-NF-κB, *p*-IκB-α, and *p*-IKKα/β were increased. Treatment with puerarin-V significantly augmented the PPAR-γ expression and suppressed the activation of *p*-NF-κB, *p*-IκB-α, and *p*-IKKα/β expression in a dose-dependent manner (Figure 7E–H). These results suggested that puerarin-V attenuated LPS-induced inflammation in HCAECs associated with upregulation of the PPAR-γ expression, and inhibition of the phosphorylation of downstream target proteins NF-κB, IκB-α and IKKα/β.

### 2.7. Puerarin-V Protected against LPS-Induced Cell Apoptosis in HCAECs

**Figure 8:**
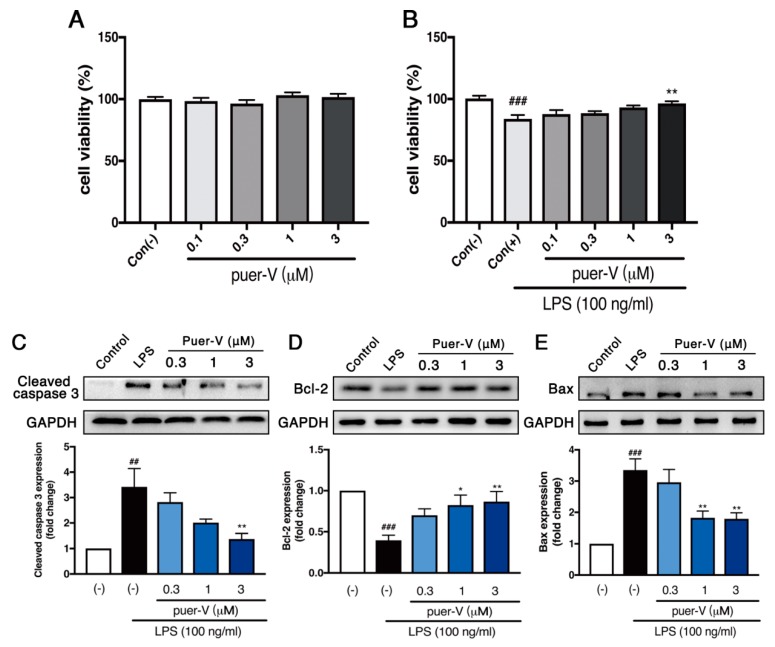
Effects of puerarin-V on cell viability and apoptosis-related protein expression in HCAECs exposed to LPS. (**A**) Effects of puerarin-V on cell viability under normaxia conditions. (**B**) Effects of puerarin-V on cell viability in LPS-induced cell injury. (**C**) Cleaved caspase 3 expression. (**D**) Bcl-2 expression. (**E**) Bax expression. Relative density analysis of the protein bands was shown by the western blot with GAPDH as control. Data are shown as mean ± SEM (*n* = 4). ^##^ *p* < 0.01, and ^###^ *p* < 0.001 vs. control group. * *p* < 0.05, and ** *p* < 0.01 vs. model group.

The potential cytotoxicity of puerarin-V and effects on LPS-induced endothelial cell injury were determined by CCK-8 assay. As depicted in Figure 8A, HCAECs were incubation with puerarin-V for 24 h, and cell viability was not significantly affected by puerarin-V at the doses used (0.1–3 μM). Therefore, we used these puerarin-V doses in this study. In addition, LPS (100 ng/mL) caused slight endothelial cell injury and cell death, whereas puerarin-V mildly protected against cellular injury in a dose-dependent manner (0.1–3 μM; Figure 8B) with the greatest effect at a dose of 3 μM.

In accordance with the results of apoptosis analysis in vivo, puerarin-V significantly downregulated the expression of cleaved caspase 3 and Bax in HCAECs exposed to LPS (Figure 8C,E). Additionally, the expression of the anti-apoptotic protein Bcl-2 was investigated. LPS prominently decreased Bcl-2 expression compared with unstimulated cells (Figure 8D), and puerarin-V significantly increased Bcl-2 expression in a dose-dependent manner. These results illustrated that puerarin-V protected HCAECs against LPS-induced apoptosis.

## 3. Discussion

Acute MI is a multifactorial and progressive disease that has not yet been fully understood. Studies on ISO-induced cardiotoxicity animal models provide a good insight into this disease and clearly indicate the involvement of inflammation and apoptosis. Therefore, we used this model to evaluate the cardioprotective effects of puerarin-V on the inflammation and apoptosis of MI in the present study.

Due to poor oral bioavailability of puerarin, puerarin injection has been widely used clinically for the treatment of cardiovascular diseases, whereas the side effects are obvious, such as pancytopenia, fever, and skin rash [20]. Moreover, previous studies demonstrated that propylene glycol, acting as a cosolvent, had great influence on puerarin injection toxicity, which pointed out that puerarin injection of toxic reactions and the amount of propylene glycol contained in them showed positive correlation [21]. Therefore, the dominant crystal was developed, called puerarin-V, which not only improved the plasma drug concentration, but also might give an opportunity to change the method of administration of puerarin. In the present study, solid intragastric administration was used to make it possible for oral administration, and the cardioprotective effects of puerarin-V and its anti-inflammatory mechanisms were studied.

In the present study, we first found that puerarin-V exerted a strong cardioprotective effects against ISO-induced MI both in vivo and in vitro. First, puerarin-V treatment decreased the incidence of mortality and inhibited the typical ECG ST segment depression at all dosages employed. Second, puerarin-V treatment effectively decreased the levels of myocardial injury markers including AST, LDH and cTn-T. Furthermore, puerarin-V treatment attenuated the process of inflammation and apoptosis in the heart, either after the ISO injection, or both before and after the ISO injection. The protective effects of puerarin-V treatment in the pre + post-ISO group seemed to be more prominent compared to those in the post-ISO group. Moreover, puerarin-V therapy upregulated PPAR-γ expression and downregulated NF-κB/IκB-α/IKKα/β phosphorylation in myocardial tissues and HCAECs, thus implying its underlying mechanisms in the MI model.

ECG is reliable for the early diagnosis of MI [22]. ST segment depression in ECG patterns was observed in ISO-induced mice compared to that in normal mice. ST segment depression is one of the characteristic manifestations of myocardial injury, which are also some of the indicative indicators of ischemia [23]. ECG abnormalities, such as ST-segment deviation, could be due to the consecutive loss of cell membrane in injured myocardium [24]. In our experiment, puerarin-V administration markedly restrained ISO-induced ST-segment depression, suggestive of its cell membrane-protecting effects. Moreover, the effects on ECG patterns of post-V and pre + post-V groups at the dose of 100 mg/kg were comparable in puerarin injection treatment. There is no difference observed between post-V and pre + post-V groups.

Myocardium contains a variety of specific enzymes, and once myocardial damage or necrosis, these enzymes are released into serum in different degrees, thus measuring the activity of these enzymes in serum to diagnose and monitor MI [25]. The study found that serum levels of AST, LDH, and cTn-T were significantly increased in ISO-treated mice, clearly suggesting that ISO-induced myocardial damage, which is consistent with previous reports [26]. Treatment with puerarin-V markedly improved the pathological elevation of these myocardial injury markers. It demonstrated that puerarin-V could maintain membrane integrity, thereby restricting the leakage of these enzymes. Notably, there was a decrease noticed in levels of these marker enzymes in the plasma of pre + post-ISO group as compared to those of post-ISO group. In other words, puerarin-V showed preventive effects on the MI model. Thus, it appears that puerarin-V could maintain membrane integrity as potent as the positive drug.

The preliminary histopathological findings of ISO-induced myocardium injury revealed marked infiltrating inflammatory cells and separation of myocardial fibers. Tissue sections from the positive group and puerarin-V groups showed varying degrees of slight inflammatory cell infiltration, diminished myocardial cell swelling, and less severe histological damage, and there was a significant difference in improvement of pathological changes between these groups. In fact, increased monocyte activation could be observed in various stages of MI. Moreover, exaggerated inflammation, tissue damage and apoptosis are usually associated with increased visible monocyte/macrophage infiltrates in the damaged myocardium [27]. In the present study, we found that puerarin-V significantly reduced the incursion of macrophages (CD68^+^ cells) into the myocardium of MI mice. Additionally, multiple studies have shown high levels of pro-inflammatory cytokines such as TNF-α, IL-6, and IL-1β in the peripheral circulation and heart of cardiac failure patients [28]. Here, we examined the changes in inflammatory cytokines in endothelial cells and myocardium. Endothelial cells form the cell layer lining the luminal surface of blood vessels and perform multiple physiological functions, such as regulating vascular tone [29], cellular growth, differentiation [30], and immune and inflammatory responses [31]. Meanwhile, endothelial cells are the targets of many endogenous and exogenous agents (e.g., LPS) that activate the endothelium and secret a variety of inflammatory mediators such as IL-1β, TNF-α, IL-6 and other neurotoxic substances [32]. There is considerable evidence that LPS contributes to the propagation of atherosclerosis, which has many features in common with inflammatory diseases [33]. In the present study, our results indicated that puerarin-V suppressed inflammatory cytokines release in vivo and in vitro experiments. Therefore, it can be speculated that puerarin-V regulates cardiac inflammation in MI mice by maintaining inflammation and anti-inflammatory balance.

Inflammation and apoptosis are crucial players in the pathogenesis of atherosclerosis, MI, and coronary heart disease [34,35]. Several studies have shown that apoptosis often occurs after expression of pro-inflammatory cytokines in MI myocardium [36]. To further investigate the possible role of apoptosis in ISO-induced MI mice, we measured apoptotic and anti-apoptotic protein expression levels. Treatment with ISO showed a significant increase in Bax expression accompanied with a decrease in Bcl-2 expression. Of note, puerarin-V abrogated this apoptotic effect as there was significantly reduced Bax expression and increased Bcl-2 expression. Moreover, treatment with puerarin-V attenuated the degree of apoptosis, measured by TUNEL detection kit. Puerarin-V reduced myocardial infarct size and cell apoptosis in heart. Additionally, these effects of puerarin-V, at least in part, are a consequence of suppression of NF-κB/IκB-α/IKKα/β, as activation of inflammatory pathway has also shown to stimulate apoptotic signaling. The present findings are in agreement with recent studies, including ours [26], suggesting that anti-apoptotic potential of puerarin-V is essential for its cardioprotective effects. Overall, the present study mainly focused on the protective effects of puerarin-V on MI mice. In other research, puerarin also exerted similar cardioprotective effects in rats, including antifibrotic effects and accelerated angiogenesis [12,13]. Therefore, the differences in such effects between puerarin and puerarin-V on MI mice will need further studies.

The present study also revealed that the myocardial salvaging effect of puerarin-V is also mediated by its anti-inflammatory effect. As predicted, in LPS-induced HCAECs injury model, we examined that puerarin-V treatment downregulated *IL-1β* and *TNF-α* gene transcripts. Further, we also observed that puerarin-V significantly inhibited the expression of IL-6 and translation activity of NF-κB/IκB-α/IKKα/β. The probable reason for this effect is inhibition of the phosphorylation of downstream target proteins NF-κB/IκB-α/IKKα/β associated with PPAR-γ activation as PPAR-γ is considered as a master regulator in inhibiting recruitment of pro-inflammatory cytokines [37]. Moreover, as illustrated in the previous literature, puerarin-V has shown to suppress inflammatory mediators including IL-6, IL-2, iNOS, NF-κB/IKK-α and COX-2 expression [38,39]. Taken together, these in vitro findings supported our hypothesis that puerarin-V protected against LPS-induced HCAECs inflammatory activity associated with activation of the PPAR-γ expression, and inhibition of pro-inflammatory cytokine production via a mechanism that involves NF-κB signaling pathway. Nevertheless, further studies needs to be carried out to use agonists and antagonists for the PPAR-γ/NF-κB signaling pathway to demonstrate the causal relationship among puerarin-V, PPAR-γ activation and cardioprotection. 

In summary, this study demonstrated that puerarin-V could prevent the development of MI in an ISO-induced mice model and alleviate myocardial injury for prevention and control. Moreover, activation of the PPAR-γ expression and inhibition of inflammation and apoptosis in heart might serve as mechanisms that are involved in the therapeutic effects of puerarin-V. Therefore, these data imply that puerarin-V may be an attractive compound for developing drugs against MI. Our research provides a new therapeutic option for MI patients.

## 4. Materials and Methods 

### 4.1. Reagents

Puerarin-V (HPLC, 98%) was provided as a lyophilized powder by the Institute of Materia Medica (Beijing, China). Puerarin injection was purchased from Beijing Shkb Pharmaceutical Co., Ltd. (Beijing, China). ISO and LPS were purchased from Sigma-Aldrich (St Louis, MO, USA). AST and LDH kits were purchased from BioSino Biotechnology & Science Inc (Beijing, China). TRIzol^®^ for total RNA extraction was purchased from Gibco (Thermo Fisher Scientific, Inc., Waltham, MA, USA). PrimeScriptTM RT Reagent Ki and SYBR^®^ Premix Ex TaqTM were purchased from TaKaRa (Shiga, Japan). The antibodies against P-NF-κBp65, NF-κBp65, p-IκB-α, IκB-α, and GAPDH were purchased from Cell Signaling Technology (Danvers, MA, USA). The antibodies against PPAR-γ, p-IKKα/β, and IKKα/β were purchased from Santa Cruz Biotechnology (Dallas, TX, USA). The antibodies against Bax, Bcl-2, and caspase 3 were purchased from Abcam (Cambridge, MA, USA). The anti-rabbit IgG or anti-mouse IgG secondary antibodies, enhanced chemiluminescence (ECL) and loading buffer were purchased from CWBIO (Beijing, China). All other chemicals used in this study are commercially available.

### 4.2. Animals and Experimental Design

Healthy male C57BL/6 mice (20–22 g, certificate No.: SCXK2016-0006) were purchased from the Vital River Laboratory Animal Center (Beijing, China) and allowed to acclimatize for 3 days in facilities, where the house temperature and humidity were controlled at 22 ± 2 °C and 45% ± 10%, respectively, with a regular 12:12 h light–dark cycle. All animals were given water and food ad libitum. All animal care and experimental procedures were reviewed and approved by the Institutional Animal Care and Use Committee of the Institute of Materia Medica, Chinese Academy of Medical Sciences and Peking Union Medical College (No. 00005553).

**Figure 9:**
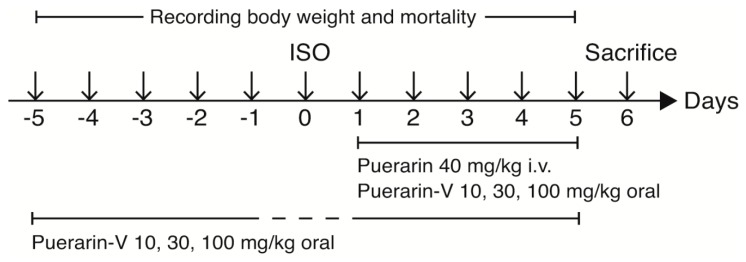
Schematic representation of animal experimental design.

The mice were randomly divided into 9 groups (Figure 9): (1) control (0.5% *w/v* aqueous CMC–Na, i.g.); (2) model group (ISO injection only); (3) positive group (puerarin injection 40 mg/kg, i.v. for 5 days after ISO injection); (4)–(6) post-ISO groups: each group administered with high, medium, and low doses of puerarin-V for 5 days after ISO injection (100, 30 and 10 mg/kg, respectively, solid i.g.); (7)–(9) pre + post-ISO groups: each group administered with high, medium, and low doses of puerarin-V for 10 days both before and after ISO injection (100, 30 and 10 mg/kg, respectively, solid i.g.) (*n* = 12 in each group). All drugs were administered once daily except day 0 on which ISO was injected. All mice were observed for body weight and mortality until day 6.

Animals were treated with ISO (40 mg/kg, s.c.) to induce experimental MI for the third time at an interval of 12 h on day 0. Mice were fasted overnight but allowed water ad libitum since the last administration of the drug.

The mice were anesthetized after the last injection of ISO, and lead II ECG was recorded continuously. ST-segment elevation or depression (expressed in mV) in experiment animals was calculated. The mice were sacrificed to analyze on the last day of experiment (day 6). Blood samples were collected from the fundus vein of the mice under anesthesia with diethyl ether. All samples were centrifuged at 4,000 rpm at 4 °C for 20 min. Serum was stored at −80 °C. The heart tissues of left ventricles were homogenized for the assays. Then, all samples of 12 mice were further randomly divided for individual experiment, excluding those which were dead during the ISO.

### 4.3. Electrocardiogram Recording

Mice were anesthetized with isoflurane (induction period: 2.5 vol.%, maintenance: 1.2 vol.%, isoflurane in 70%N_2_O/30%O_2_) and peripheral limb electrodes were inserted subcutaneously. ECG was recorded at 24 h before the MI model was established, as well as 1 and 5 days after the MI model was established. ECG waveforms were inspected visually for rhythm and configuration abnormalities. ECG parameters (ST segment and T wave) were generated using the BL-420S Biologic Function Experiment system (Chengdu, China).

### 4.4. TTC Staining

Following determination of cardiac function outcome measurements, the mice were sacrificed and their hearts were dissected and washed repeatedly with cold N.S. The connective tissue on the surface of the heart was removed, and then serial sections were cut. The sections were stained with 1% TTC solution for 20 min in the dark in a shaking water bath with a constant temperature (37 °C). The sections were photographed.

### 4.5. Determination of AST, LDH and cTn-T Release into Serum 

Myocardial cellular injury was evaluated by measuring serum AST and LDH levels. At the end of the experiment, the serum AST and LDH activities were measured spectrophotometrically, and serum cTn-T was quantified using the enzyme-linked immunosorbent assay (ELISA) kit (cTn-T; Cusabio, Inc., Wuhan, China) according to the manufacturer′s instructions.

### 4.6. Measurement of Inflammatory Cytokines by ELISA

The left ventricles of the mice were homogenized with a homogenizer and then centrifuged at 5000 rpm for 15 min at 4 °C. The expression of IL-6, IL-1β, and TNF-α in the supernatant were measured using ELISA kits (IL-6, IL-1β, and TNF-α; Cusabio, Inc., Wuhan, China) according to the manufacturer′s instructions. Briefly, anti-mouse antibody was coated on the ELISA plate, and 100 μL of testing samples or standard were added to each well and incubated for 2 h at 37 °C. The biotinylated anti-mouse antibody solution was added to each well incubated for 1 h at 37 °C. After incubation, liquid was discarded and washed 3 times repeatedly. After it dried, the horseradish peroxidase (HRP)–streptavidin-conjugated secondary antibody was added to each well and incubated for 1 h at 37 °C. After washing, TMB solution was added and incubated at room temperature. Finally, a stop solution was added to each well and absorbance at 450 nm was measured using a microplate reader. The cytokine concentration was determined using a standard curve.

### 4.7. H&E Staining and Immunohistochemical Analysis of Macrophage Marker CD68

The hearts were washed immediately with saline and then fixed in 4% buffered paraformaldehyde solution. Tissues were embedded in paraffin, sectioned at 5 m and stained with H&E) [40] and primary antibody CD68 [41]. These sections were examined under a light microscope for histoarchitectural changes, and then photomicrographs were taken. Semi-quantification of CD68^+^ cells within a total tissue area in the microscopic field was obtained for 6 random fields in cardiac tissues using Image Pro Plus 5.1 (Media Cybernetics, Bethesda, MD, USA).

### 4.8. Western Blot Analysis

Total cell or mice heart tissue lysates (20–50 μg) were subjected to SDS-PAGE. Electrophoresis and blotting were performed as described [17]. Immunoreactivity bands were visualized by enhanced chemiluminescence (ECL) and captured through a ChemiDoc-lTR 510 image system (Upland, CA, USA). For quantification, ECL signals were digitized using Quantity One (Bio-rad, Hercules, CA, USA).

### 4.9. Total RNA Extraction for Quantitative Real-Time Polymerase Chain Reaction (PCR)

Total RNA was isolated from the heart tissue or HCAECs using the TRIzol reagent, followed by reverse transcription from 1 μg of total RNA to cDNA, and then, cDNA was synthesized using a PrimeScriptTM RT reagent Kit, followed by real-time PCR using an SYBR^®^ Premix Ex Taq according to the manufacturer’s instructions. The PCR reaction for mRNA, except human tumor necrosis factor-α (hTNFα), was performed in triplicate with the following conditions: 95 °C for 30 s, then 40 cycles of 95 °C for 5 s, and 60 °C for 30 s. For hTNFα, the PCR conditions was carried out with the following conditions: 95 °C for 2 min, then 40 cycles of 95 °C for 30 s, 56 °C for 30 s and 72 °C for 30 s, and the final extension was performed at 72 °C for 5 min on the CFX96TM Real-time System (Bio-Rad, Singapore) and repeated at least three times. The samples were matched to a standard curve generated by amplifying serially diluted products using the same real-time PCR conditions. The data were presented as the fold change in mRNA expression levels and were normalized by the mRNA expression levels of an endogenous reference gene *GAPDH*. Primer sequences were provided in Table 1.

### 4.10. Determination of Myocardial Apoptosis

A TUNEL assay was performed using In Situ Cell Death Detection kit according to the manufacturer′s instructions. Both positive (DNase-treated) and negative (no addition of terminal transferase) control tissue sections were incorporated into each assay. Individual nuclei were visualized at 200× magnification, and the percentage of TUNEL-positive nuclei (positive nuclei/total nuclei) was calculated using Image Pro Plus 5.1 from at least six randomly chosen fields per slide for statistical analysis. All of these measurements were performed in a blinded manner.

### 4.11. HCAEC Culture

HCAECs were obtained from ScienCell Research Laboratories (Carlsbad, CA, USA). Cells were cultured in Minimum Essential Medium (MEM; Gibco, St. Katharinen, Germany) supplemented with 15% of fetal bovine serum (FBS) (Gibco, Australia) in humidified air at 37 °C with 5% CO_2_. After reaching confluence, cells were detached from culture flasks using Trypsin–EDTA, washed and resuspended in a complete medium. All experiments were done with cells kept in culture between three and six passages.

### 4.12. Cell Viability Assay

The toxicity of puerarin-V and/or LPS on HCVEC cells was determined by Cell Counting Kit-8 (CCK-8, Dojindo, Japan) assay according to the manufacturer′s instructions. Briefly, following the above cell treatment protocol, cells were seeded in 96-well plates with a density of 5 × 10^3^ cells per well and cultured in minimum essential medium (MEM) containing 15% FBS for 24 h, and the medium was then replaced with a serum-free medium for 24 h. Serum-starved cells were incubated for 24 h with 100 ng/mL LPS and/or puerarin-V at concentrations between 0.1 and 3 μM. One hundred microliters of CCK-8 (diluted 1:10) solution were added to each well followed by incubation with 5% CO_2_ at 37 °C for 1.5 h. Absorbance was measured at 450 nm using a Molecular Devices SpectraMax M5 microplate reader. Cell viability was expressed as a percentage of the untreated control.

### 4.13. Stimulation of Cells with LPS and IL-6 Measurements

HCAECs were seeded in 100 μL complete medium with a density of 5 × 10^3^ cells per well in 96-well plates or in a 2 mL complete medium with a density of 8 × 10^4^ cells per well in 6-well plates. After growing to confluency and then being synchronized by serum deprivation for 24 h, synchronized cells were treated with different concentrations of puerarin-V for 2 h and then stimulated with LPS (100 ng/mL) for 24 h. The supernatants were collected for IL-6 measurement using a Human IL-6 ELISA kit (Thermo Fisher Scientific, Vienna, Austria) according to the protocols given by the manufacturers. All experiments were repeated at least three times with different preparations of endothelial cells.

### 4.14. Statistical Analysis

All data were expressed as the mean ± standard error of the mean (SEM) and analyzed by GraphPad Prism 7 (GraphPad Software, San Diego, CA, USA). Statistical analysis was performed using one-way analysis of variance (ANOVA) followed by Dunnett′s post hoc test. The effect of puerarin-V on the survival of mice was analyzed by the Kaplan–Meier methods and compared by the log-rank test. A *p*-value < 0.05 was considered as statistically significant.

## Figures and Tables

**Table 1:** Primers sequences used in real-time PCR analysis. F: forward; R: reverse.

mRNA			Primer Sequence	
hTNF-α ^1^	F	5′	ATGAGCACTGAAAGCATGATC	3′
	R	5′	TCACAGGGCAATGATCCCAAAGTAGACCTGCCC	3′
hIL-6 ^2^	F	5′	GCCTTCGGTCCAGTTGCCTT	3′
	R	5′	AGTGCCTCTTTGCTGCTTTCAC	3′
hIL-1β	F	5′	CAGCCATGCCAGAAGTACCT	3′
	R	5′	GACATCACCAAGCTTTTTTGC	3′
hGAPDH	F	5′	CCACCCATGGCAAATTCCATGGCA	3′
	R	5′	TCTAGACGGCAGGTCAGGTCCACC	3′
mTNF-α	F	5′	GGCTGCCCCGACTACGT	3′
	R	5′	AGGTTGACTTTCTCCTGGTATGAGA	3′
mIL-6	F	5′	TTCCATCCAGTTGCCTTCTTG	3′
	R	5′	GGGAGTGGTATCCTCTGTGAAGTC	3′
mIL-1β	F	5′	CTACAGGCTCCGAGATGAACAAC	3′
	R	5′	TCCATTGAGGTGGAGAGCTTTC	3′
mGAPDH ^3^	F	5′	TGCACCACCAACTGCTTAGC	3′
	R	5′	GGCATGGACTGTGGTCATGAG	3′

^1^ hTNF-α: human tumor necrosis factor-α, ^2^ hIL-6: human interleukin 6, ^3^ mGAPDH: mouse glyceraldehyde-3-phosphate dehydrogenase.

## References

[1] Allawadhi P., Khurana A., Sayed N., Kumari P., Godugu C. (2018). Isoproterenol-induced cardiac ischemia and fibrosis: Plant-based approaches for intervention. Phytother. Res..

[2] Liu Y.T., Zhou C., Jia H.M., Chang X., Zou Z.M. (2016). Standardized Chinese Formula Xin-Ke-Shu inhibits the myocardium Ca(2+) overloading and metabolic alternations in isoproterenol-induced myocardial infarction rats. Sci. Rep..

[3] Raish M. (2017). Momordica charantia polysaccharides ameliorate oxidative stress, hyperlipidemia, inflammation, and apoptosis during myocardial infarction by inhibiting the NF-κB signaling pathway. Int. J. Biol. Macromol..

[4] Liu J., Sui H., Zhao J., Wang Y. (2017). Osmotin Protects H9c2 Cells from Simulated Ischemia-Reperfusion Injury through AdipoR1/PI3K/AKT Signaling Pathway. Front. Physiol..

[5] Fan D., Yang Z., Yuan Y., Wu Q.Q., Xu M., Jin Y.G., Tang Q.Z. (2017). Sesamin prevents apoptosis and inflammation after experimental myocardial infarction by JNK and NF-κB pathways. Food Funct..

[6] Olefsky J.M., Glass C.K. (2010). Macrophages, inflammation, and insulin resistance. Annu. Rev. Physiol..

[7] Qi H.P., Wang Y., Zhang Q.H., Guo J., Li L., Cao Y.G., Li S.Z., Li X.L., Shi M.M., Xu W. (2015). Activation of peroxisome proliferator-activated receptor gamma (PPARgamma) through NF-κB/Brg1 and TGF-beta1 pathways attenuates cardiac remodeling in pressure-overloaded rat hearts. Cell Physiol. Biochem..

[8] Lv F.H., Yin H.L., He Y.Q., Wu H.M., Kong J., Chai X.Y., Zhang S.R. (2016). Effects of curcumin on the apoptosis of cardiomyocytes and the expression of NF-κB, PPAR-gamma and Bcl-2 in rats with myocardial infarction injury. Exp. Ther. Med..

[9] Rani N., Bharti S., Bhatia J., Nag T.C., Ray R., Arya D.S. (2016). Chrysin, a PPAR-gamma agonist improves myocardial injury in diabetic rats through inhibiting AGE-RAGE mediated oxidative stress and inflammation. Chem. Biol. Interact..

[10] Cui J., Wang G., Kandhare A.D., Mukherjee-Kandhare A.A., Bodhankar S.L. (2018). Neuroprotective effect of naringin, a flavone glycoside in quinolinic acid-induced neurotoxicity: Possible role of PPAR-gamma, Bax/Bcl-2, and caspase-3. Food Chem. Toxicol..

[11] Zhou Y.X., Zhang H., Peng C. (2014). Puerarin: A review of pharmacological effects. Phytother. Res..

[12] Cai S.A., Hou N., Zhao G.J., Liu X.W., He Y.Y., Liu H.L., Hua Y.Q., Li L.R., Huang Y., Ou C.W. (2018). Nrf2 Is a Key Regulator on Puerarin Preventing Cardiac Fibrosis and Upregulating Metabolic Enzymes UGT1A1 in Rats. Front. Pharmacol..

[13] Ai F., Chen M., Yu B., Yang Y., Xu G., Gui F., Liu Z., Bai X., Chen Z. (2015). Puerarin accelerate scardiac angiogenesis and improves cardiac function of myocardial infarction by upregulating VEGFA, Ang-1 and Ang-2 in rats. Int. J. Clin. Exp. Med..

[14] Cheng W., Wu P., Du Y., Wang Y., Zhou N., Ge Y., Yang Z. (2015). Puerarin improves cardiac function through regulation of energy metabolism in Streptozotocin-Nicotinamide induced diabetic mice after myocardial infarction. Biochem. Biophys. Res. Commun..

[15] Rajadurai M., Stanely Mainzen Prince P. (2007). Preventive effect of naringin on cardiac markers, electrocardiographic patterns and lysosomal hydrolases in normal and isoproterenol-induced myocardial infarction in Wistar rats. Toxicology.

[16] Huang H., Geng Q., Yao H., Shen Z., Wu Z., Miao X., Shi P. (2018). Protective effect of scutellarin on myocardial infarction induced by isoprenaline in rats. Iran. J. Basic. Med. Sci..

[17] Gong L.L., Fang L.H., Wang S.B., Sun J.L., Qin H.L., Li X.X., Wang S.B., Du G.H. (2012). Coptisine exert cardioprotective effect through anti-oxidative and inhibition of RhoA/Rho kinase pathway on isoproterenol-induced myocardial infarction in rats. Atherosclerosis.

[18] Iqbal A.J., McNeill E., Kapellos T.S., Regan-Komito D., Norman S., Burd S., Smart N., Machemer D.E., Stylianou E., McShane H. (2014). Human CD68 promoter GFP transgenic mice allow analysis of monocyte to macrophage differentiation in vivo. Blood.

[19] Neri T., Armani C., Pegoli A., Cordazzo C., Carmazzi Y., Brunelleschi S., Bardelli C., Breschi M.C., Paggiaro P., Celi A. (2011). Role of NF-κB and PPAR-gamma in lung inflammation induced by monocyte-derived microparticles. Eur. Respir. J..

[20] Jiang H. (2008). Literature analysis of 35 cases of acute hemolytic anemia due to puerarin. Tianjin Pharm..

[21] Zhou Y.B., Pan W.S. (2010). The importance of pharmaceutical excipients according to the abnormal toxicity of puerarin. Chin. Med. Her..

[22] Wang J.J., Pahlm O., Warren J.W., Sapp J.L., Horacek B.M. (2018). Criteria for ECG detection of acute myocardial ischemia: Sensitivity versus specificity. J. Electrocardiol..

[23] Reinstadler S.J., Baum A., Rommel K.P., Eitel C., Desch S., Mende M., Metzler B., Poess J., Thiele H., Eitel I. (2015). ST-segment depression resolution predicts infarct size and reperfusion injury in ST-elevation myocardial infarction. Heart.

[24] Mohler P.J., Schott J.J., Gramolini A.O., Dilly K.W., Guatimosim S., duBell W.H., Song L.S., Haurogne K., Kyndt F., Ali M.E. (2003). Ankyrin-B mutation causes type 4 long-QT cardiac arrhythmia and sudden cardiac death. Nature.

[25] Awad M.A., Aldosari S.R., Abid M.R. (2018). Genetic Alterations in Oxidant and Anti-Oxidant Enzymes in the Vascular System. Front. Cardiovasc. Med..

[26] Guo J., Wang S.B., Yuan T.Y., Wu Y.J., Yan Y., Li L., Xu X.N., Gong L.L., Qin H.L., Fang L.H. (2013). Coptisine protects rat heart against myocardial ischemia/reperfusion injury by suppressing myocardial apoptosis and inflammation. Atherosclerosis.

[27] Reddy S.S., Agarwal H., Barthwal M.K. (2018). Cilostazol ameliorates heart failure with preserved ejection fraction and diastolic dysfunction in obese and non-obese hypertensive mice. J. Mol. Cell Cardiol..

[28] Glezeva N., Baugh J.A. (2014). Role of inflammation in the pathogenesis of heart failure with preserved ejection fraction and its potential as a therapeutic target. Heart Fail. Rev..

[29] Ketsawatsomkron P., Sigmund C.D. (2015). Molecular mechanisms regulating vascular tone by peroxisome proliferator activated receptor gamma. Curr. Opin. Nephrol. Hypertens..

[30] Zhang Z., Chen Y., Zhang T., Guo L., Yang W., Zhang J., Wang C. (2016). Role of Myoendothelial Gap Junctions in the Regulation of Human Coronary Artery Smooth Muscle Cell Differentiation by Laminar Shear Stress. Cell Physiol. Biochem..

[31] Zhaocheng J., Jinfeng L., Luchang Y., Yequan S., Feng L., Kai W. (2016). Ginkgolide A inhibits lipopolysaccharide-induced inflammatory response in human coronary artery endothelial cells via downregulation of TLR4-NF-κB signaling through PI3K/Akt pathway. Pharmazie.

[32] Chen X., Zhang Y., Wang W., Liu Z., Meng J., Han Z. (2018). Mesenchymal Stem Cells Modified with Heme Oxygenase-1 Have Enhanced Paracrine Function and Attenuate Lipopolysaccharide-Induced Inflammatory and Oxidative Damage in Pulmonary Microvascular Endothelial Cells. Cell Physiol. Biochem..

[33] Lin G., Shi X., Chen S., Lei L., You X., Huang M., Luo L., Li Y., Zhao X., Yan F. (2015). Effects of micro-amounts of Porphyromonas gingivalis lipopolysaccharide on rabbit inflammatory immune response and development of atherosclerosis. J. Periodontal. Res..

[34] Li X., Deroide N., Mallat Z. (2014). The role of the inflammasome in cardiovascular diseases. J. Mol. Med..

[35] Kang P.M., Izumo S. (2003). Apoptosis in heart: Basic mechanisms and implications in cardiovascular diseases. Trends Mol. Med..

[36] Zhang J., Liao Y., Cheng X., Chen J., Chen P., Gao X., Zhang Z. (2006). Myosin specific-T lymphocytes mediated myocardial inflammation in adoptive transferred rats. Cell Mol. Immunol..

[37] Dang X., Du G., Hu W., Ma L., Wang P., Li Y. (2018). Peroxisome proliferator-activated receptor gamma coactivator-1alpha/HSF1 axis effectively alleviates lipopolysaccharide-induced acute lung injury via suppressing oxidative stress and inflammatory response. J. Cell Biochem..

[38] Hou B., Zhao Y., Qiang G., Yang X., Xu C., Chen X., Liu C., Wang X., Zhang L., Du G. (2018). Puerarin Mitigates Diabetic Hepatic Steatosis and Fibrosis by Inhibiting TGF-beta Signaling Pathway Activation in Type 2 Diabetic Rats. Oxid. Med. Cell Longev..

[39] Tu Y.M., Gong C.X., Ding L., Liu X.Z., Li T., Hu F.F., Wang S., Xiong C.P., Liang S.D., Xu H. (2017). A high concentration of fatty acids induces TNF-alpha as well as NO release mediated by the P2X4 receptor, and the protective effects of puerarin in RAW264.7 cells. Food Funct..

[40] Chen Y.C., Yuan T.Y., Zhang H.F., Wang D.S., Yan Y., Niu Z.R., Lin Y.H., Fang L.H., Du G.H. (2016). Salvianolic acid A attenuates vascular remodeling in a pulmonary arterial hypertension rat model. Acta. Pharmacol. Sin..

[41] Vorkapic E., Lundberg A.M., Mayranpaa M.I., Eriksson P., Wagsater D. (2015). TRIF adaptor signaling is important in abdominal aortic aneurysm formation. Atherosclerosis.

